# Enhanced photoelectrocatalytic degradation of diclofenac sodium using a system of Ag-BiVO_4_/BiOI anode and Ag-BiOI cathode

**DOI:** 10.1038/s41598-022-08213-0

**Published:** 2022-03-10

**Authors:** Benjamin O. Orimolade, Omotayo A. Arotiba

**Affiliations:** 1 Department of Chemical Sciences, University of Johannesburg, Johannesburg, South Africa; 2 Centre for Nanomaterials Science Research, University of Johannesburg, Johannesburg, South Africa

**Keywords:** Environmental sciences, Materials science, Chemistry, Electrochemistry, Environmental chemistry, Surface chemistry

## Abstract

We report the photoelectrocatalysis of diclofenac sodium using a reactor consisting of Ag-BiVO_4_/BiOI anode and Ag-BiOI cathode. The electrodes were prepared through electrodeposition on FTO glass and modified with Ag nanoparticles through photodeposition. The structural and morphological studies were carried out using XRD, SEM, and EDS which confirmed the successful preparation of the materials. The optical properties as observed with UV-DRS revealed that the electrodes were visible light active and incorporation of metallic Ag particles on the surface increased the absorption in the visible light region. Presence of p-n heterojunction in the anode led to decrease in the spontaneous recombination of photoexcited electron–hole pairs as seen in the photocurrent response. The results from photoelectrocatalytic degradation experiments revealed that replacing platinum sheet with Ag-BiOI as counter electrode resulted in higher (92%) and faster removal of diclofenac sodium as evident in the values of apparent rate constants. The reaction mechanism further revealed that efficiently separated photogenerated holes played a major role in the degradation of the pharmaceutical. The prepared electrodes showed good stability and impressive reusability. The reports from this study revealed that the dual photoelectrodes system has a great potential in treating pharmaceutical polluted wastewater using visible light irradiation.

## Introduction

Water stress due to water pollution has remained a major global problem over the recent years. Among the vast groups of water pollutants, much attention has been channeled to the removal of residual pharmaceuticals which belong to the group known as emerging contaminants from wastewater^1^. Residual pharmaceuticals originating from pharmaceutical industries, hospitals and household effluents often end up in water bodies and hence have been detected in groundwater and surface water^2^. The presence of pharmaceuticals in surface water above the permitted no effect concentration (PNEC) is harmful to aquatic organisms^3^. Likewise, continuous intake of pharmaceutical contaminated water by humans could have adverse effect such as endocrine disruption, hindering cell regeneration and antibiotics resistance^4,5^. Therefore, it is important to completely remove such pharmaceuticals from polluted water. However, pharmaceuticals are highly soluble in water and very difficult to remove making conventional wastewater treatment plants unsuitable to completely eliminate them from wastewater. In the recent years, much focus has been on the application of advanced oxidation techniques which are capable of complete mineralisation of organics for the removal pharmaceuticals in aqueous solutions^6–8^.

Among the numerous forms of AOPs, photoelectrocatalytic (PEC) oxidation/degradation of organics has been identified to be efficient in breaking down of a wide spectrum of organic pollutants. In PEC degradation, both light and electrical energy are used to drive the oxidation of organics in aqueous media^9,10^. The principle of PEC degradation is similar to that of typical photocatalysis in that the photoanodes in PEC system are often made up of semiconductor photocatalysts. However, unlike in typical photocatalysis, applied bias potential in PEC degradation significantly reduces the problem of spontaneous recombination of photogenerated electron–hole pairs because the potential provides sufficient force that creates electric field within the space-charge layer of the semiconductor which promote separation of charge carriers to the substrate and electrons can eventually be driven away from the photoanode or the semiconductor. Another interesting advantage of PEC is lower energy consumption when compared to anodic oxidation which requires high potential/current to facilitate degradation of organic^11^. Several kinds of semiconductors, particularly TiO_2_ and ZnO, have been employed as electrode materials in PEC degradation^12,13^. However, in order limit the cost associated with PEC degradation, visible light sources are currently being employed to excite the semiconductor photoanodes. This implies that the semiconductors themselves should be visible light active. Examples of commonly used visible light active semiconductors are BiVO_4_^14^, g-C_3_N_4_^15^, Fe_2_O_3_^16^, WO_3_^17,18^, Cu_2_O^19^ and MoS_2_^20^. It is worth noting that as a result of the band gap and appropriate and edge positions, BiVO_4_ has become a very popular candidate for anodic material in PEC applications. Nevertheless, the experimental PEC efficiency of BiVO_4_ photoanodes are still below its theoretical efficiency^21,22^. This is largely due to the problem of rapid recombination of photogenerated charge carriers. The formation of heterojunction of BiVO_4_ with other suitable semiconductor has proven to be an efficient method for promoting charge separation within BiVO_4_ photoanodes. Hence, heterostructures of photoanodes of BiVO_4_/Bi_2_O_3_^23^, BiVO_4_/ZnO^24^, BiVO_4_/Ag_2_S^25^, BiVO_4_/Ag_3_PO_4_^26^, and BiVO_4_/WO_3_^27^ have shown better PEC efficiency than pristine BiVO_4_ photoanode.

Furthermore, the PEC efficiency of BiVO_4_ can be improved through the addition of noble metals (gold, silver and platinum) on its surface. This is because there are several free vibrating electrons on the surface of such noble metals which oscillate after being irradiated with light of suitable frequency resulting in generation of electronic wave called localized surface plasmon resonance (LSPR) effect^28,29^. Therefore, noble metals can act as photosensitizers promoting the absorption of visible light. Additionally, noble metals can form Schottky barrier when in contact with semiconductors when the electrons from the interface of the semiconductor move towards the noble metal^30,31^. This largely promote the separation of photogenerated charge carriers because electrons from the conduction band of the semiconductors can easily migrate to the conduction levels of the noble metals. For instance, Wu et al*.* reported enhanced photocatalytic efficiency of Ag decorated BiVO_4_ and it was observed that Ag widens the visible light response range of BiVO_4_ and also facilitates enhanced charge separation of photogenerated electron–hole pairs which resulted in higher removal of tetracycline^32^. Similarly, in the works of Li et al*.*, it was reported that the photodeposited Ag on BiVO_4_/MnO_x_ formed a local magnetic field in synergy with heterojunction electric field through plasmonic resonance effect and this significantly improved charge separation resulting in better photocatalytic performance^33^.

In this present study, Ag-BiVO_4_/BiOI photoanode prepared through photodeposition of metallic Ag on the surface of BiVO_4_/BiOI heterojunction was employed in the PEC degradation of diclofenac sodium salt, a representative pharmaceutical compound that has been detected in the environment. Earlier, BiVO_4_/BiOI heterostructured photoanode has been established to promote efficient charge separation of photogenerated electron hole pairs through the formation of Type II p-n heterojunction^34^. However, the presence of metallic Ag on the surface of BiVO_4_/BiOI can further promote charge separation within the charge separation and improve visible light absorption leading to better PEC efficiency. Additionally, in order to reduce the cost and energy consumption associated with PEC degradation systems, Ag-BiOI is employed as counter electrode in lieu of platinum sheet. The use of Ag-BiVO_4_/BiOI photoanode with Ag-BiOI counter is not only capable of reducing cost, but also enhance PEC efficiency due to unequal Fermi energy level of the two electrodes which also promote charge separation. Therefore, the dual photoelectrodes PEC system in this work has a great potential for oxidation of pharmaceuticals with minimal cost. The details of the effect of PEC experimental parameters such as inner electrode distance and bias potential were also investigated.

## Materials and methods

### Preparation of electrodes

#### Preparation of Ag-BiVO_4_/BiOI photoanode

A previously reported electrodeposition method (with slight modifications) was employed for the preparation of BiOI, BiVO_4_ and BiVO_4_/BiOI films on FTO glass (50 mm × 13 mm × 2.2 mm, surface resistivity of ~ 7 Ω/sq)^34^. Firstly, BiOI films on FTO were formed via electrodeposition. This was achieved by sonicating a mixture of 0.49 g Bi(NO_3_)_3_·5H_2_O and 1.66 g KI in 25 mL deionized for 20 min. To this solution, 10 mL of 0.23 M p-benzoquinone dissolved in absolute ethanol was added. The solution was further sonicated for 15 min and the pH was adjusted to 4 by the addition of 275 μL of 1 M NaOH. Electrodeposition was performed at a potential of − 0.13 V for 300 s. FTO glass, platinum wire and Ag/AgCl (3.0 M KCl) electrode were employed as the working electrode, counter electrode and reference electrode respectively. The as-prepared BiOI electrodes were rinsed with deionised water several times. A 100 μL of 0.20 M vanadylacetylacetonate (dissolved in DMSO) was drop-cast onto the BiOI electrode. The electrode was then heated in a furnace at 420 °C for 1 h. Finally, the electrode was immersed in 1.0 M NaOH solution for 30 min to remove excess V_2_O_5_. The resulting BiVO_4_/FTO electrode was washed with deionized water and dried at room temperature. Subsequent electrodeposition of BiOI onto the BiVO_4_/FTO produced the BiVO_4_/BiOI electrode.

The Ag-BiVO_4_/BiOI photoanode was obtained through photodeposition of Ag. This was done by dipping the electrodeposited BiVO_4_/BiOI in a methanolic solution (50% v/v) of 0.1 M AgNO_3_ in the dark for 30 min under continuous stirring which was followed by irradiation with a 100 W Xenon lamp (equivalent of 1.3 sun) for specified duration^32^. The samples were designated 1Ag-BiVO_4_/BiOI, 2Ag-BiVO_4_/BiOI and 3Ag-BiVO_4_/BiOI which corresponds to irradiation time of 15 min, 30 min and 45 min respectively. The 2Ag-BiVO_4_/BiOI which showed the best photocurrent response was selected for all other experiments and analyses.

#### Preparation of Ag-BiOI

The electrodeposited BiOI on FTO was dipped into the solution of 0.01 M AgNO_3_ in the dark for 30 min under continuous stirring and was followed by irradiation with 100 W Xenon lamp for 30 min.

### Structural and morphology characterisation of the prepared electrodes

X-ray diffraction patterns of the prepared electrodes were obtained on X-ray diffractometer (Rigaku Ultima IV, Japan) using Cu Kα radiation (k = 0.15406) with K-beta filter at 30 mA and 40 kV. TESCAN Vega 3 (Czech Republic) scanning electron microscope coupled with energy-dispersive X-ray spectrometer (EDS) was used to examine the surface morphologies as well as elemental compositions of the materials. The optical properties of the materials were observed by recording using UV–visible diffuse reflectance spectroscopy on Cary 60 UV–vis spectrophotometer (Malaysia) with barium sulphate as the reflectance.

### Electrochemical and photoelectrochemical experiments

Autolab 302 N potentiostat/galvanostat was employed for all electrochemical measurements using a three-electrode configuration. Ag/AgCl (3.0 M KCl) was used as the reference electrode while platinum foil was used as the counter electrode when necessary. The fabricated electrodes were employed as the working electrodes. The light source for photoelectrochemical experiments was a solar simulator (100 W xenon lamp). The prepared electrodes were positioned vertically with the anode facing the solar simulator and the distance between the working electrode and the light source was kept constant at 10 cm while the distance between the working electrode and counter electrode was maintained at 2 cm. For the experiments with Ag-BiVO_4_/BiOI as anode and Ag-BiOI as cathode, the Ag-BiVO_4_/BiOI was placed facing the light (Control experiments were also conducted with platinum sheet facing the light). Photocurrent measurements and linear sweep voltammetry were carried out in a 0.1 M Na_2_SO_4_ solution. A 5 mM solution of [Fe(CN)_6_]^3−/4−^ (prepared in a 0.1 M KCl solution) was used for electrochemical impedance spectroscopy measurements. In the case of the PEC degradation experiments, 10 mg L^−1^ of diclofenac sodium with 0.1 M solution of Na_2_SO_4_ as supporting electrolyte was used and the experiments were carried out in a 50 mL quartz reactor. At given time intervals, aliquots were collected from the reactor. The change in concentration of the pharmaceutical over time was recorded with the aid of UV–Visible spectrophotometer while the extent of mineralisation was evaluated by measuring the total organic carbon on a TOC analyser (Teledyne Tekmar TOC fusion). The effects of inner electrode distance and applied bias potential on the PEC removal efficiency of the system were investigated.

## Results and discussion

### Structural, optical and morphology characterisation of the electrodes

**Figure 1:**
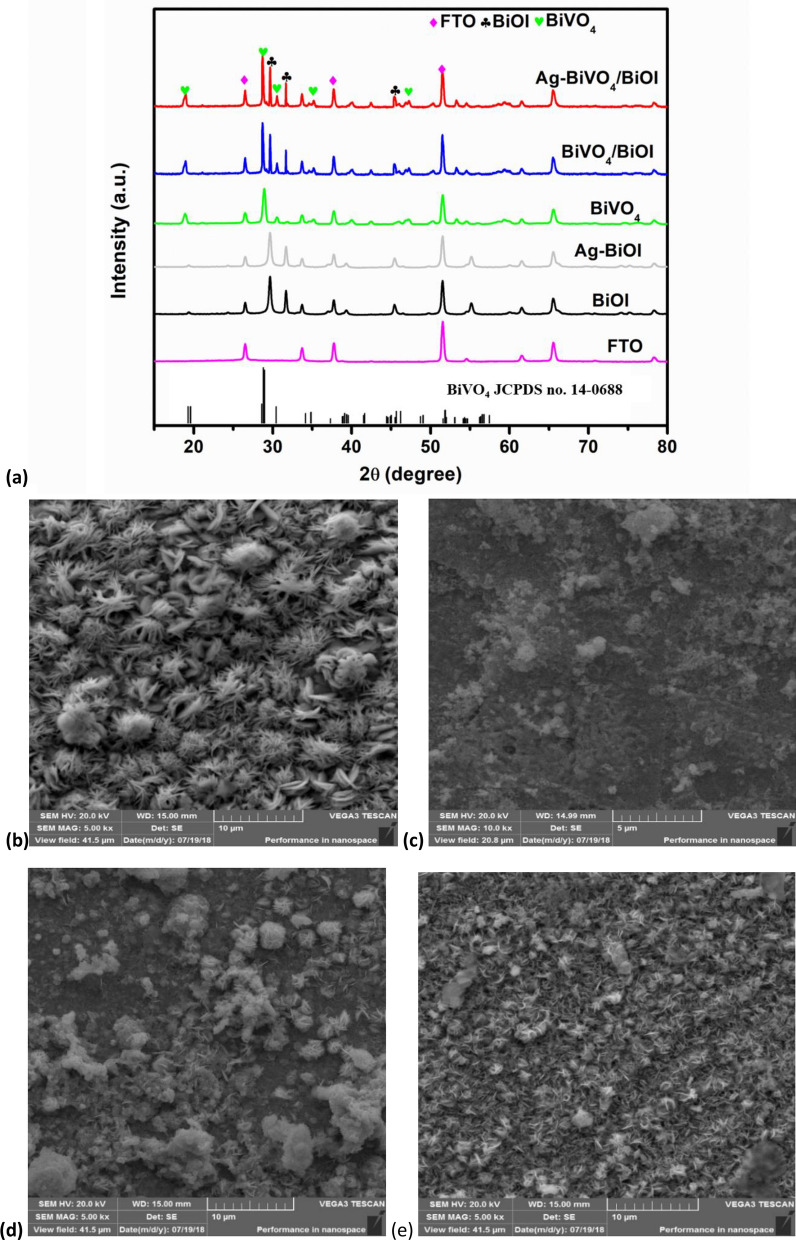

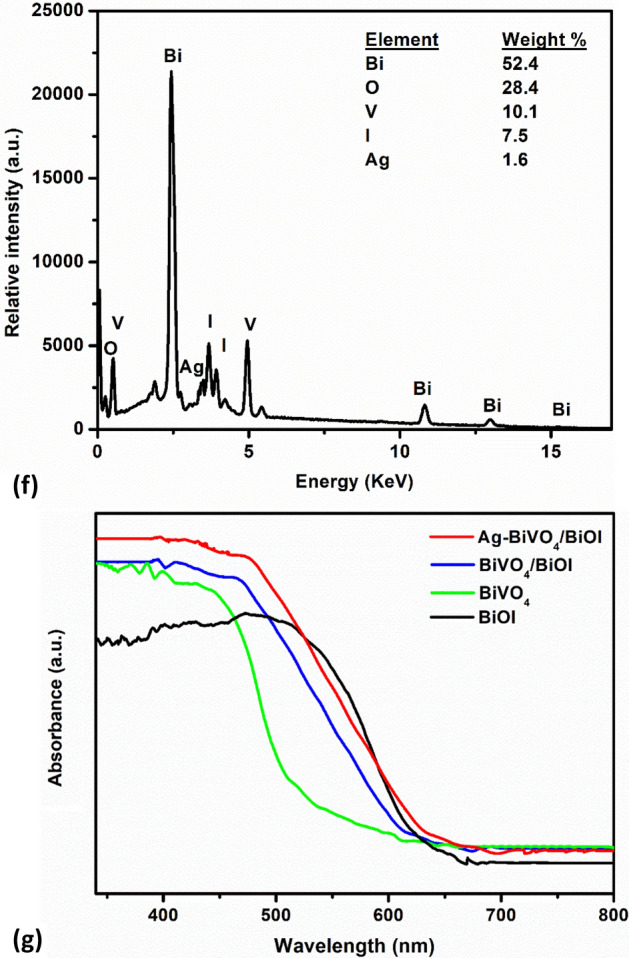
(**a**) XRD pattern of BiOI, Ag-BiOI, BiVO_4_, BiVO_4_/BiOI and Ag-BiVO_4_/BiOI; SEM images of (**b**) BiOI, (**c**) BiVO_4_, (**d**) BiVO_4_/BiOI and (**e**) Ag-BiVO_4_/BiOI; (**f**) EDS spectrum of Ag-BiVO_4_/BiOI; (**g**) UV–Vis DRS spectra of BiOI, BiVO_4_, BiVO_4_/BiOI and Ag-BiVO_4_/BiOI.

The X-ray diffractograms of the prepared photoanodes are shown in Fig. 1. The distinct peaks observed in the XRD pattern of BiOI are at 29.67°, 32.01° and 45.27° which correspond to the (102), (110) and (200) planes of tetragonal BiOI (JCPDS no. 10-445)^35^. All the peaks of BiOI remained obvious in the XRD pattern of Ag-BiOI with no additional peak of metallic Ag and this could be due to low content of Ag in the material. Similar trend has been reported with BiOI modified with Ag quantum dots^36^. In the case of the prepared BiVO_4_, the major peaks in the diffractogram are at 18.4°, 29.02°, 30.71°, 33.91°, 35.15°, 40.01° and 42.62° corresponding to the (110, 011), (121), (040), (200), (002), (211) and (150) planes of monoclinic scheelite BiVO_4_ respectively (JCPDS no. 75-1866)^37^. The BiVO_4_/BiOI photoanode contained all the characteristics peaks of BiVO_4_ and BiOI which suggest a successful preparation of the composite electrode. Just as in the case of Ag-BiOI, only the major peaks of BiVO_4_ and BiOI are visible in the XRD pattern of Ag-BiVO_4_/BiOI with no Ag peak and this could also be ascribed to the low content of Ag in the composite material. Similar observations have been reported in XRD patterns of semiconductors modified with noble metals^36,38^. Other characterisation techniques were used to establish the presence of Ag in the materials.

The surface morphologies of the prepared materials as observed through SEM images can be seen in Fig. 1b–e. The BiOI electrodeposited on FTO glass possessed consistent flower-like microstructures of different sizes whereas the BiVO_4_ particles existed as irregular agglomerated structures on the FTO glass. Subsequent electrodeposition of BiOI on the FTO-BiVO_4_ electrode led to the incorporation of the particles of BiVO_4_ within the openings of BiOI which consequently reduced the agglomeration of BiVO_4_ particles as seen in the micrograph of BiVO_4_/BiOI. Interestingly, the loading of Ag on the composite electrode remarkably reduced the agglomeration of the particles. The TEM images of the materials showed that Ag nanoparticles surrounds the BiVO4/BiOI particles (Fig. S1). To further established the presence of the Ag on the BiVO_4_/BiOI, EDS analysis of the Ag-BiVO_4_/BiOI was carried out and it was observed that Ag in addition to Bi, V, O and I was present with no other impurities (Fig. 1f). However, the percentage of Ag (< 1%) in the bulk material is low when compared to the other elements. Nevertheless, the EDS mapping (Fig. S2) clearly revealed that Ag particles are well dispersed on the surface of the electrode.

Since the prepared semiconductors are visible light active, the absorption of photons within the visible light region by the prepared photoanodes was investigated through UV/Vis–diffuse reflectance spectroscopy. From the results presented in Fig. 1g, it was evident that all the prepared materials absorbed light within the visible region. Specifically, the absorption edges were 671, 532, 654 and 685 nm for BiOI, BiVO_4_, BiVO_4_/BiOI and Ag-BiVO_4_/BiOI respectively. The formation of p-n heterojunction within BiVO_4_ and BiOI coupled with the presence of Ag particles greatly extend the absorption edge of Ag-BiVO_4_/BiOI towards the visible light region and also increased its absorbance. Furthermore, the band gap energies of the BiOI and BiVO_4_ were calculated using the Tauc’s plot (Fig. S3) and the values were 1.92 eV and 2.34 eV for BiOI and BiVO_4_ respectively. These values are in agreement with both the theoretical and reported values for the band gap energies of the semiconductors^39,40^.

### Electrochemical and Photoelectrochemical characterisation

**Figure 2:**
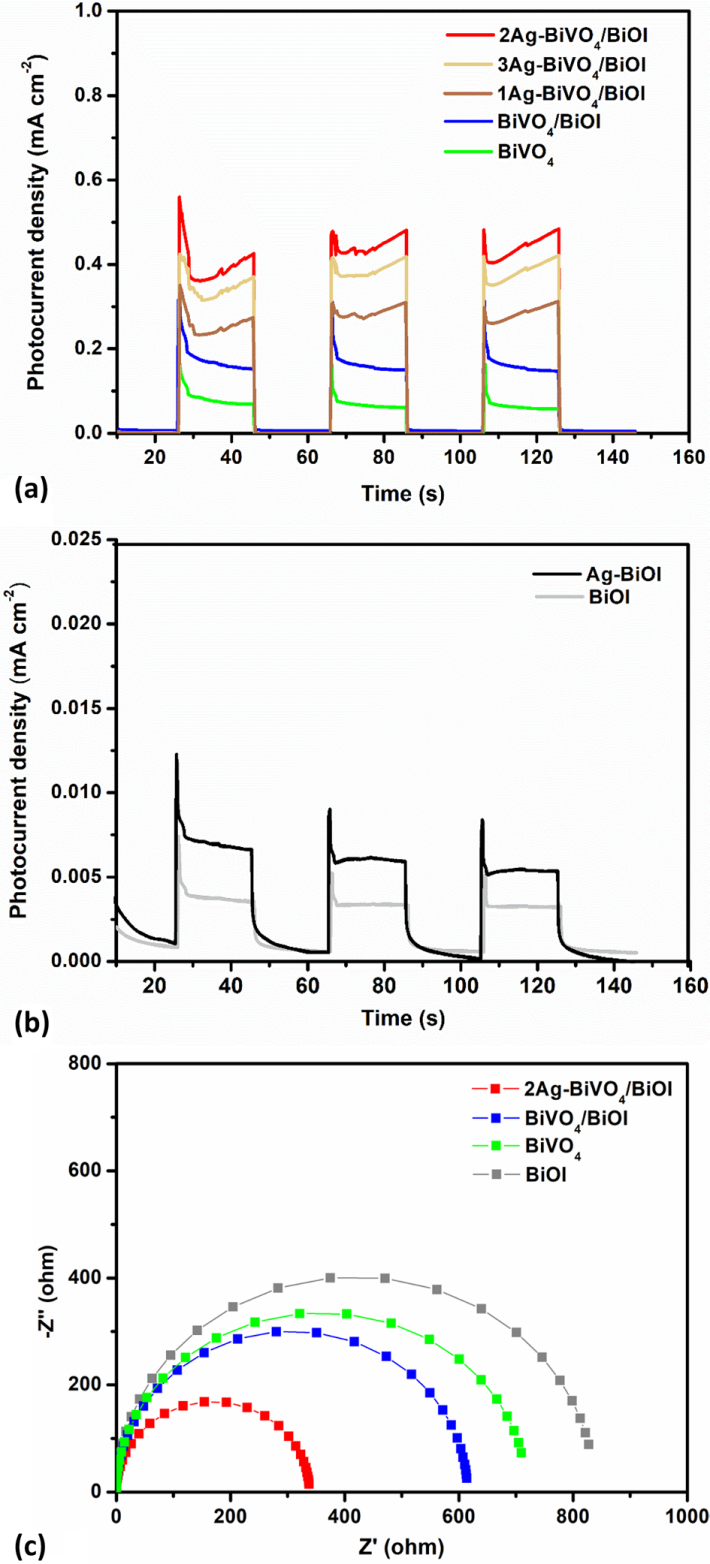
Photocurrent responses of (**a**) photoanodes BiVO_4_, BiVO_4_/BiOI and Ag-BiVO_4_/BiOI at 0.5 V vs Ag/AgCl in 0.1 M Na_2_SO_4_ (pH 7); (**b**) photocathodes BiOI, Ag-BiOI; and (**c**) EIS spectra of the materials in 5 mM [Fe(CN)_6_]^−3/−4^ in 0.1 M KCl.

Measurements of transient photocurrent responses of the prepared photoanode was carried out in 0.1 M Na_2_SO_4_ with an applied bias of + 0.5 V to investigate the charge separation happening within the constructed heterojunction. As presented in Fig. 2a,b, all the materials responded well when irradiated with visible light. The magnitude of the photocurrent response recorded with BiVO_4_/BiOI was 0.34 mA cm^−2^ which was about twice of that of BiVO_4_ (0.18 mA cm^−2^) and largely higher than that of BiOI (0.0072 mA cm^−2^). This revealed that the formation of p-n heterojunction resulted in efficient separation of photogenerated electron–hole pairs which consequently promote better photon harvesting in the composite electrode. Loading of Ag particles on the composite electrode further improve the charge separation efficiency within the heterostructured photoanodes.

The photodeposition time of Ag has a significant effect on the photocurrent response. From Fig. 2a where 1Ag-BiVO_4_/BiOI, 2Ag-BiVO_4_/BiOI and 3Ag-BiVO_4_/BiOI represents Ag deposition time of 15 min, 30 min and 45 min respectively, it can be seen that increasing the deposition time from 15 to 30 min gave enhanced photocurrent response. However, further increase in the deposition time to 45 min led to a decrease in the magnitude of the photocurrent response. This can be ascribed to the fact that, excess amount of Ag can saturate the surface of the electrode and thereby preventing photons to sufficient reach the semiconductors. Nevertheless, all the Ag loaded photoanodes gave better photocurrent response and the highest response was 0.58 mA cm^−2^. This observation clearly affirmed that as a noble metal, Ag could act as a photosensitizer absorbing photons and regulating the generation of charge carrier through direct electron transfer or dipole–dipole coupling connection^31,41^. Additionally, the behaviour of the electrodes with different bias potential was also examined through LSV with light on/off and it was observed that the magnitude of the transient photocurrent response increased with the applied potential (Fig. S4).

To further understand the charge-transfer properties of the composite photoanode, electrochemical impedance spectroscopy was performed with bias potential of + 0.2 V. The obtained Nyquist plots are presented in Fig. 2c. Information regarding the charge-transfer resistance (R_ct_) at the interface of the heterojunction can be obtained from the semi-circular arc. Generally, studies have confirmed that the smaller the arc diameter the higher the charge transfer efficiency^42,43^. Thus, the arc diameter of the Nyquist plots of the electrodes are in the order of Ag-BiVO_4_/BiOI < BiVO_4_/BiOI < BiVO_4_ < BiOI. The Ag-BiVO_4_/BiOI with the least R_ct_ value (113 Ω) revealed that the electron mobility within the material is highest and this can be attributed to the presence of metallic Ag which further promote efficient charge separation within the BiVO_4_/BiOI p-n heterojunction.

Furthermore, the change in carrier density and flat band potential can provide insight into the efficient charge separation within the composite electrode. Therefore, Mott Schottky plots (Eq. 1) were made to investigate the shift in the flat band potential and the carrier density of the heterojunction.

1 $$ 1/C^{2} = 2/(e\varepsilon \varepsilon_{0} N_{D} ) \cdot (E_{app} - E_{FB} - KT/e) $$

where C, e, ɛ, ɛ_0_, N_D_, E_app_, E_FB_, k and T are the capacitance at the semiconductor/electrolyte interface (Fcm^-2^), elementary charge (1.60 × 10^–19^ C), dielectric constant (68 for BiVO_4_^44^), permittivity of vacuum, donor density, applied potential (V), flat band potential, Boltzmann constant and absolute temperature respectively. Donor density (N_D_) was calculated from the slope of the plot of 1/C^2^ versus E_app_ while the flat band potential was obtained from the intercept (Fig. S5). A positive slope value was obtained in the MS plot for BiVO_4_ indicating an n-type semiconductor while a negative slope was observed in that of BiOI being p-type semiconductor. It was further observed that the value of E_FB_ for BiVO_4_ decreased from − 0.637 to − 0.693 V in the BiOI/BiVO_4_. Upon the addition of Ag, the flat band potential further decreased to − 0.713. This negative shift is attributed to the inhibition of recombination of the photogenerated electron–hole pairs. This observation was further justified by the calculated values of charge carrier densities with Ag-BiVO_4_/BiOI possessing the highest (8.66 × 10^22^ cm^−3^) than both BiOI/BiVO_4_ (3.31 × 10^22^ cm^−3^) and BiVO_4_ (6.24 × 10^21^ cm^−3^). Negative shift of flat band potential has also been reported in Ag doped semiconductors and this is due to the fact that Ag can act as an electron donor and hence it facilitates efficient charge separation within the Ag-doped overlayer on the surface of the semiconductor^45^.

### PEC Degradation of diclofenac sodium

**Figure 3:**
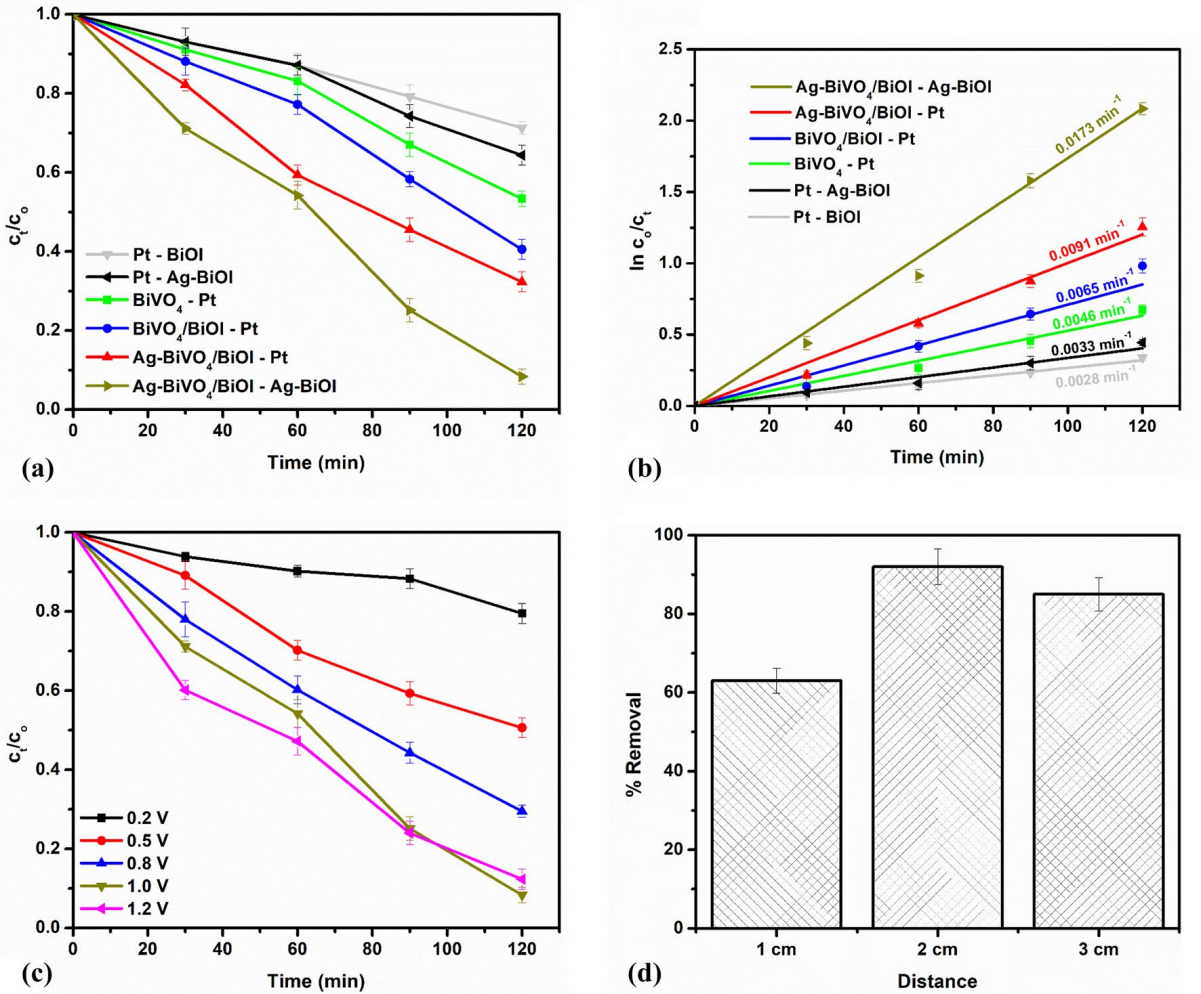
(**a**) Normalized concentration decay pattern for the PEC degradation of diclofenac sodium using the prepared photoanodes and photocathodes; (**b**) corresponding kinetics plot; Effect of (**c**) applied bias potential, (**d**) inner electrode distance on the PEC degradation process with Ag-BiVO_4_/BiOI–Ag-BiOI system (Co = 10 mg L^−1^; pH 7; 1.0 V bias potential).

The prepared electrodes were employed for the PEC degradation of 10 mg L^−1^ diclofenac sodium with an applied bias of 1.0 V (vs Ag/AgCl) under simulated solar light using defined configurations and UV/Vis spectrophotometer was used to monitor the concentration changes. In the cases of the photoanodes with platinum sheet as counter electrode (photoanode—Pt), the Ag-BiVO_4_/BiOI achieved 68% removal which was higher than 59% and 46% recorded with BiVO_4_/BiOI and BiVO_4_ photoanodes respectively (Fig. 3a). The percentage removal with BiVO_4_/BiOI was higher than BiVO_4_ due to the formation of p-n heterojunction between BiVO_4_ and BiOI which promoted better charge separation and consequently reduced recombination of electron–hole pairs. The presence of metallic Ag particles in Ag-BiVO_4_/BiOI significantly improved its PEC performance by promoting electron mobility, better visible light absorption and efficient separation of photogenerated electron–hole pairs through accelerated interfacial electron transfer by the formation of Schottky barrier which resulted in better percentage removal. In previous reports, researchers have also achieved enhanced degradation efficiencies of organics by incorporating Ag particles into BiVO_4_ and other heterostructures^32,46^. The synergistic effect of light and applied potential in the PEC degradation process using the Ag-BiVO_4_/BiOI was also investigated and it was observed that both the photocatalytic degradation and electrochemical oxidation resulted in far less percentage removal of analyte (Fig. S6). Combining the two processes in the PEC resulted in better performance because the applied bias potential provided a driving force which facilitate the movement of the photogenerated electrons from the photoanode surface thereby minimizing rapid recombination of charge carriers.

In order to further improved the PEC degradation of diclofenac sodium using Ag-BiVO_4_/BiOI photoanode and to significantly reduce the cost associated with the PEC process through the use of expensive platinum sheet counter electrode, Ag-BiOI was employed as a counter electrode (Ag-BiVO_4_/BiOI—Ag-BiOI). Interestingly, the percentage PEC removal of the pharmaceutical increased tremendously to about 92% after 2 h (Fig. 3a). It is worth noting that when Ag-BiOI was used as a photocathode with platinum sheet as anode (Pt—Ag-BiOI), the percentage removal was only 34% (When the Pt was facing the light source, the percentage removal was only 21% Fig. S7). Therefore, combining the prepared photoanode with the photocathode is suitable and efficient for the PEC removal of diclofenac sodium. Additionally, the TOC removal in the combined electrode system was 63% which was far greater than 41% achieved with using the photoanode with platinum sheet counter electrode. From the TOC values, the specific energy consumptions, EC_TOC_ (kWh g^−1^ of TOC), of the processes were calculated using Eq. (2).

2 $$ {\text{EC}}_{{{\text{TOC}}}} = \frac{{{\text{VIt}}}}{{(\Delta {\text{TOC}})_{\exp } {\text{V}}_{{\text{S}}} }} $$

where the applied potential (V), average current (A), reaction time (h) and solution volume (L) are denoted as V, I, t and V_s_ respectively. In the case of the Ag-BiVO_4_/BiOI photoanode with platinum sheet, the energy consumption was 1.217 kWh g^−1^ of TOC. However, upon substitution of the platinum sheet, the energy consumption decreased to 0.928 kWh g^−1^ of TOC. The reduction in the energy consumption revealed that the combined process is more energy efficient.

The kinetics study further deepened the understanding of the PEC degradation processes. The experimental data were fitted into linearised Langmuir–Hinshelwood kinetics model (ln c_o_/c_t_ = kt). Where ‘k’ is the apparent rate constant which was obtained from the slope of the plot. As shown in Fig. 3b, the PEC degradation process combining Ag-BiVO_4_/BiOI photoanode and Ag-BiOI photocathode has the highest apparent rate constant of 0.0173 min^−1^ which reveal that the process is relatively fast. On the other hand, the PEC process with Ag-BiVO_4_/BiOI photoanode and platinum counter has a lesser apparent rate constant of 0.0091 min^−1^ while the Ag-BiOI photocathode with platinum counter possessed a far lesser of 0.0033 min^−1^. Form the values obtained, it is therefore necessary to establish if combining the two electrodes resulted in a cumulative effect or a synergistic effect and this was done by calculating the degree of process synergy, S, using Eq. (3).

3 $$ S = (K_{Dual} - (K_{PA} + K_{PC} ))/K_{Dual} $$

where K_*Dual*_ is the apparent rate constant of the PEC process using Ag-BiVO_4_/BiOI—Ag-BiOI system. K_*PA*_ is the apparent rate constant of PEC system of Ag-BiVO_4_/BiOI photoanode with platinum counter while K_*PC*_ is the apparent rate constant of Ag-BiOI photocathode with platinum counter. Accordingly, the calculated degree of process synergy (S) was 0.28. The value of S is greater than zero revealed that the using the prepared Ag-BiVO_4_/BiOI photoanode with Ag-BiOI resulted in a synergistic effect and not a cumulative effect.

The efficiency of photoelectrocatalytic degradation process greatly depends on the magnitude of applied bias potential. Effect of bias potential on the degradation system was studied using a range of 0.2–1.2 V (vs Ag/AgCl). As shown in Fig. 3c, increase in applied potential led to increase in the percentage removal from 0.2 to 1.0 V. This is because bias potential provides a driving force that facilitates the migration of photogenerated electrons away from the photoanode surface towards the cathode which consequently reduces recombination of electron–hole pairs on the photoanode surface. The higher the bias potential within a lower range, the higher the driving force and hence improved PEC efficiency. However, with application of higher potential of 1.2 V, an increase in removal was observed within an hour but after that no further increase was observed. This revealed that the charge separation efficiency due to applied bias potential can reach an over saturation point with elevated potential and that higher applied bias potential could result in insignificant improvement in the percentage degradation due to side reaction of oxygen evolution from water splitting at higher potential which greatly hinders the degradation process^47^. In a previous study on the PEC degradation of diclofenac sodium, a similar trend was reported whereby increase in the applied bias potential from 1.0 to 1.5 V (vs SCE) also resulted in no remarkable increase in the degradation efficiency^48^. Moreover, application of higher potential is not energy efficient and could affect the integrity of the electrodes. Based on the result obtained, 1.0 V was selected as the optimal bias potential for the PEC degradation of diclofenac sodium in this study.

Another important parameter that affects photoelectrochemical process is the inner electrode spacing. This was studied by varying the distance between the photoanode and cathode within the range of 1–3 cm while keeping all other working parameters constant (Fig. 3d). Highest PEC removal of the pharmaceutical was observed at distance of 2 cm. At a farther distance of 3 cm, a reduction in the percentage removal was observed which can be due to large ohmic drop within the electrolyte^49^. However, the lowest removal was recorded at distance of 1 cm. A shorter inner electrode distance often lead to excessive current and this has been reported to cause oxygen/hydrogen evolution at the surface of photoanode in PEC applications and consequently result in reduced efficiency^50^.

The stability and reusability of the electrodes in the dual PEC system was also assessed through cycling experiments. This was carried out by making use of the electrodes for six consecutive cycles and after each run/cycle, the electrodes were simply rinsed and reused. As presented in Fig. S8, the electrodes displayed good reusability even after the sixth cycle since the difference in the percentage removal is less than 5%. Additionally, XRD analysis of the electrodes after the repeated use were done and as seen in Fig. S9, the characteristic peaks of the semiconductors were still present in both the anode (Ag-BiVO_4_/BiOI) and cathode (Ag-BiOI) which further establish the good stability and reusability of the electrode. The material displayed good stability as result of many factors. Firstly, unlike in photocatalysis where harsh chemicals are often used to regenerate material after use, in this case the material was simple rinsed with deionized after each use since it was used in form of a compact electrode. Additionally, the PEC degradation condition was favourable because a low bias potential (1.0 V) which could not cause the electrode to leach was applied.

### Scavenger studies and proposed mechanism

**Figure 4:**
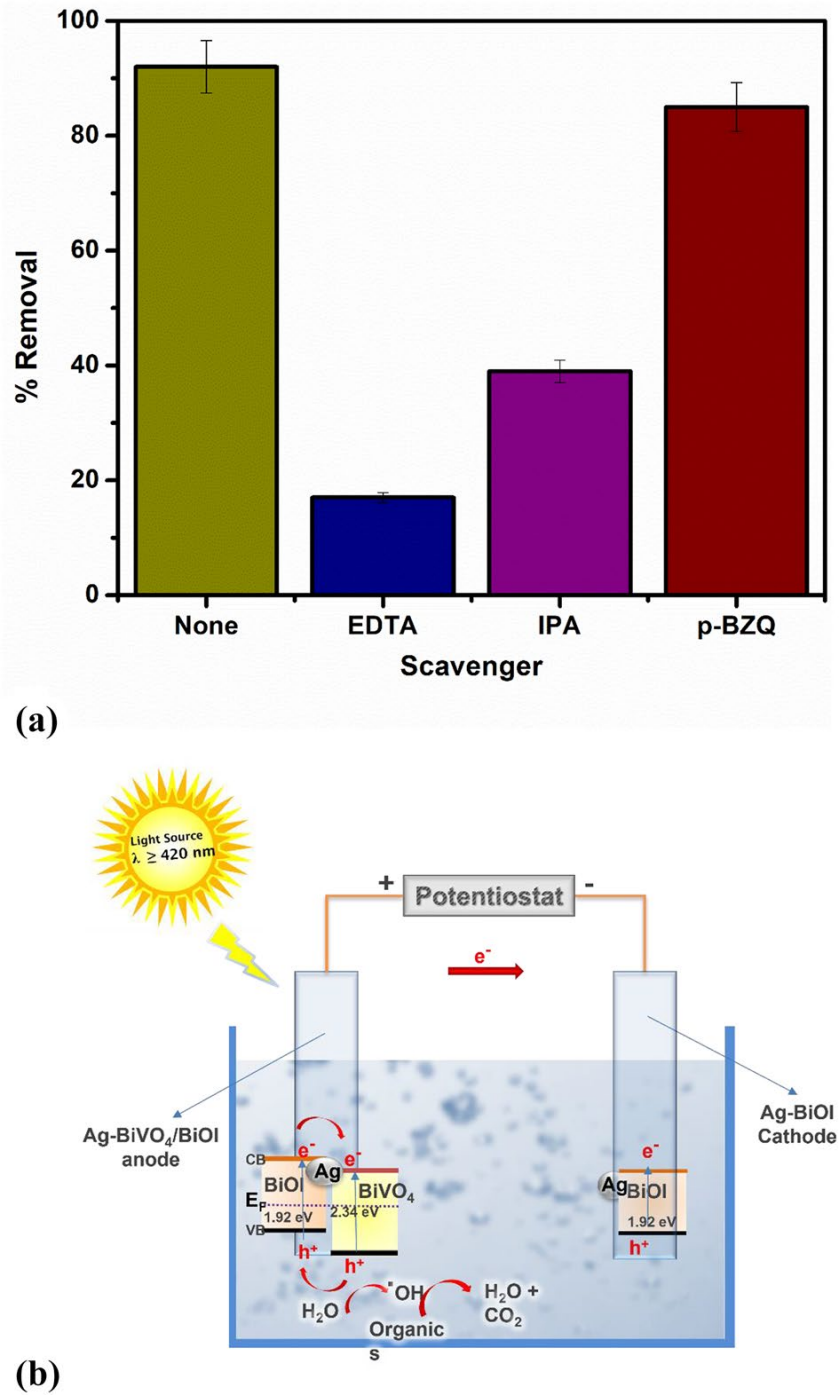
(**a**) Scavenger studies on the PEC degradation of diclofenac sodium; (**b**) proposed mechanism of the PEC degradation process in the dual electrode system.

Generally, in PEC degradation process, several reactive species are generated that can oxidize organic molecules. Such common reactive species include photogenerated holes, hydroxyl radicals and superoxide radicals. The specific contributions of each of these species were assessed by performing trapping experiments. This was done through the additions of 0.001 M Isopropanol (IPA), 0.02 M p-benzoquinone (p-BQ) and 0.01 M sodium ethylenediaminetetraacetate (EDTA) into the PEC system to mask the contributions of hydroxyl radicals, superoxide radicals and holes respectively^25,51,52^. As shown in Fig. 4a, when EDTA was added into the system, the percentage removal of diclofenac sodium dropped sharply from 92% to around 27%. This substantial change in the percentage removal indicated that photogenerated holes contributed significantly to the PEC oxidation of the diclofenac sodium molecules. In the presence of IPA, a pronounced decrease in the percentage removal was also observed from 92 to 39%, which suggests that hydroxyl radicals produced from the reaction of photogenerated holes with water molecules also play a major role in the degradation process. On the other hand, we recorded no marked change in the percentage removal when p-BZQ was present in the reaction medium. From these findings, we infer that the well separated photogenerated holes in the photoanode play the predominant role in the degradation process.

The mechanism of efficient charge separation within the Ag-BiVO_4_/BiOI photoanode, as depicted in Fig. 4b, can further be understood by considering the role of the p-n heterojunction between p-BiOI and n-BiVO_4_ as well as the junction formed with metallic Ag. In this regard, the relative band edge potentials of conduction band and valence band are determined using Eqs. (4) and (5).

4 $$ E_{CB} = X - E_{c} - 0.5E_{g} $$

5 $$ E_{VB} = E_{g} + E_{CB} $$

E_CB_ and E_VB_ are the conduction and valence band edge potentials respectively. X is the electronegativity of the semiconductor usually rendered in as the geometric mean of the absolute electronegativities of the constituents atoms (X = 6.04 eV for BiVO_4_ and 6.10 eV for BiOI^53,54^). E_C_ is the energy of the free electrons on hydrogen scale which is approximately 4.50 eV. E_g_ is the band gap potential of the semiconductors which has been estimated to 2.34 eV for BiVO_4_ and 1.92 eV for BiOI. Therefore, the E_CB_ and E_VB_ of BiVO_4_ were calculated to be 0.37 eV and 2.71 eV respectively while for BiOI, the values were 0.64 eV and 2.56 eV for E_CB_ and E_VB_ respectively. Additionally, being a p-type semiconductor, the Fermi energy level of BiOI is located slightly above the valence band while that of BiVO_4_ is located below the conduction band edge. Therefore, when the two materials are combined, an internal electric field is formed by the migrations of electron until the Fermi energy levels are aligned and the direction of the electric field is from BiVO_4_ to BiOI. Consequently, photogenerated electrons can easily migrate from the conduction band of BiOI to the conduction band of BiVO_4_ while the photogenerated holes in the valence band of BiVO_4_ migrate to the valence band of BiOI and thereby leading to efficient charge separation. Upon the addition plasmonic Ag, after excitation, a local magnetic field is created and electrons from BiVO_4_ can further migrate into space charged region of Ag, thereby enhancing separation of photogenerated electron hole pairs^33^. Additionally, it has been established that metallic noble metals are capable of improving the visible light absorption of semiconductors through interfacial electron transfer^55^. Therefore, the improved performance of the photoanode in the present study could not be solely attributed to suppression of rapid recombination of electron–hole pairs. Moreover, the use of Ag-BiOI as counter favours the migration of electrons from the photoanode towards the photocathode due to mismatch Fermi energy level^56^. The mechanism of the PEC cell explained herein is shown in the schematics in Fig. 4b. Consequently, well-separated photogenerated holes are readily available to oxidise the diclofenac molecules directly or to oxidise water molecules to produce hydroxyl radicals that also breaks down the organic molecules (pollutants).

## Conclusion

In this study, the efficiency of BiVO_4_/BiOI photoanode was successfully enhanced through the photodeposition of Ag metal on its surface. The prepared Ag-BiVO_4_/BiOI absorbed more photons in the visible light region than the heterostructured BiVO_4_/BiOI photoanode which was attributed to interfacial electron transfer by the formation of Schottky barrier. Likewise, the novel photoanode showed higher photocurrent response and better PEC degradation performance. Furthermore, the PEC degradation performance of the photoanode was further improved by adopting Ag decorated BiOI as a counter electrode in place of traditional platinum sheet. Overall, the 92% PEC removal of diclofenac sodium was achieved with the dual photo electrode system and this was significantly higher than what was recorded using platinum sheet as counter electrode. It is also interesting to note that the use of Ag-BiOI as counter electrode for the Ag-BiVO_4_/BiOI photoanode reduced the energy consumption in the PEC system. The mechanism of the PEC process revealed that photogenerated holes played the predominant role in degradation process which was as a result of efficient charge separation due to formation of type II p-n heterojunction, presence of the Ag and unequal Fermi energy levels between the semiconductor anode and cathode. The PEC system constructed in this work has a great potential for PEC degradation of pharmaceuticals with desired efficiency and at a low cost.

## Supplementary Information

Supplementary Information.
