# Supplementary material for: Enhanced photoelectrocatalytic degradation of diclofenac sodium using a system of Ag-BiVO4/BiOI anode and Ag-BiOI cathode
Source: Sci Rep. 2022 Mar 10;12:4214. doi: 10.1038/s41598-022-08213-0 (PMC8913733; doi:10.1038/s41598-022-08213-0)
Supplement: Supplementary file 1 — Supplementary Information. [file 41598_2022_8213_MOESM1_ESM.docx]

**Enhanced photoelectrocatalytic degradation of diclofenac sodium using a system of Ag-BiVO_4_/BiOI anode and Ag-BiOI cathode**

Benjamin O. Orimolade^a^, Omotayo A. Arotiba^a,b*^

*^a^Department of Applied Chemistry, University of Johannesburg, South Africa*

*^b^Centre for Nanomaterials Science Research, University of Johannesburg, South Africa*

**Corresponding author: Email addresses: oarotiba@uj.ac.za*

**Supplementary Figures**

**(a)**
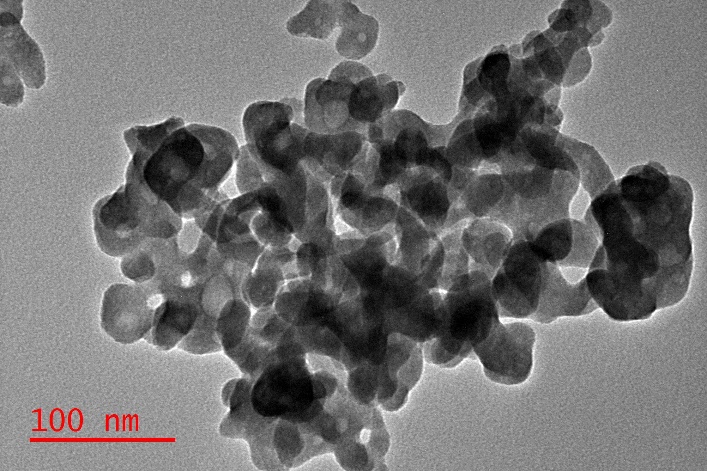


**(b)**
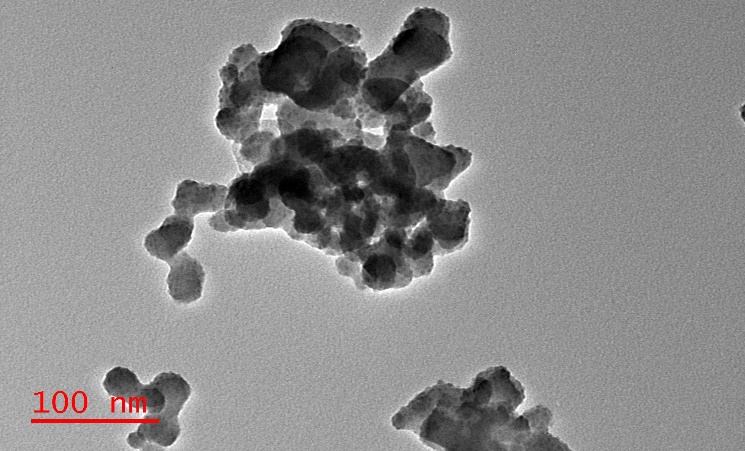


**Figure S1: TEM images of (a) BiVO_4_/BiOI and (b) Ag-BiVO_4_/BiOI**


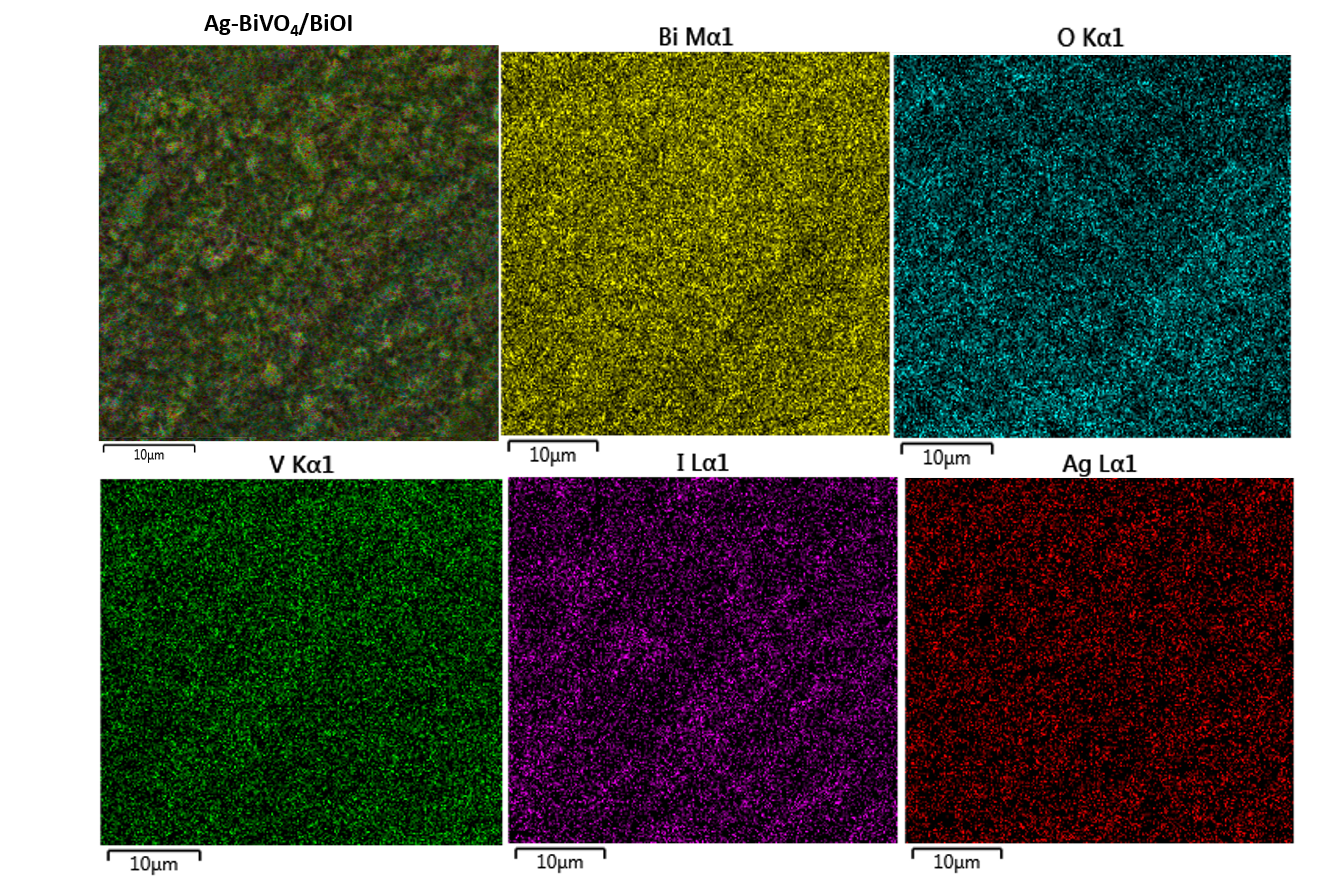


**Figure S2: EDS mapping of Ag-BiVO_4_/BiOI**





**Figure S3: Tauc’s plot showing the band gap energies of prepared BiOI and BiVO_4_.**





**Figure S4: Linear Sweep voltammograms with light ON/OFF in 0.5 M Na_2_SO_4_ at scan rate of 0.01 mVs^-1^**





**Figure S5: Mott Schottky plots for BiOI, BiVO_4_, BiVO_4_/BiOI and Ag-BiVO_4_/BiOI electrodes in 5 mM [Fe(CN)_6_]^-3/-4^ in 0.1 M KCl at pH 7**





**Figure S6: Photocatalysis, electrochemical oxidation and photoelectrocatalytic degradation of diclofenac sodium using Ag-BiVO_4_/BiOI electrode (1.0 V; pH 7; 10 mgL^-1^; 0.1M Na_2_SO_4_)**





**Figure S7: Photoelectrocatalytic degradation of diclofenac sodium using Ag-BiOI electrode with light incident on Ag-BiOI, light incident on platinum foil (Pt – Ag-BiOI (B)) and in the absence of light (Pt – Ag-BIOI (Dark)) (1.0 V; pH 7; 10 mgL^-1^; 0.1 M Na_2_SO_4_)**





**Figure S8: Reusability test of PEC system of Ag-BiVO_4_/BiOI anode and Ag-BiOI cathode.**





**Figure S9: XRD spectrum of Ag-BiVO_4_/BiOI anode and Ag-BiOI cathode before use and after use.**
